# Interrelation between Neuroendocrine Disturbances and Medical Complications Encountered during Rehabilitation after TBI

**DOI:** 10.3390/jcm4091815

**Published:** 2015-09-22

**Authors:** Caroline I. E. Renner

**Affiliations:** Neurological Rehabilitation Centre, University of Leipzig, Muldentalweg 1, D-04828 Bennewitz bei Leipzig, Germany; E-Mail: renner@sachsenklinik.de or caroline.renner@herzog-julius-klinik.de; Tel.: +49-3425-888-497; Fax: +49-3425-888-877

**Keywords:** gender, injury severity, partial pituitary insufficiency, hormonal influences, sex differences, severe traumatic brain injury

## Abstract

Traumatic brain injury is not a discrete event but an unfolding sequence of damage to the central nervous system. Not only the acute phase but also the subacute and chronic period after injury, *i.e.*, during inpatient rehabilitation, is characterized by multiple neurotransmitter alterations, cellular dysfunction, and medical complications causing additional secondary injury. Neuroendocrine disturbances also influence neurological outcome and are easily overlooked as they often present with diffuse symptoms such as fatigue, depression, poor concentration, or a decline in overall cognitive function; these are also typical sequelae of traumatic brain injury. Furthermore, neurological complications such as hydrocephalus, epilepsy, fatigue, disorders of consciousness, paroxysmal sympathetic hyperactivity, or psychiatric-behavioural symptoms may mask and/or complicate the diagnosis of neuroendocrine disturbances, delay appropriate treatment and impede neurorehabilitation. The present review seeks to examine the interrelation between neuroendocrine disturbances with neurological complications frequently encountered after moderate to severe TBI during rehabilitation. Common neuroendocrine disturbances and medical complications and their clinical implications are discussed.

## 1. Introduction

Traumatic brain injury (TBI) is a critical public health and socio-economic problem throughout the world [1]. It is a major cause of lifelong disability [2]. Patients having sustained moderate to severe TBI experience significant impairments, including motor, sensory, cognitive, emotional, and behavioural concerns, even years post injury [3].

The International Mission on Prognosis and Analysis of randomized Controlled Trials in TBI (IMPACT) has validated the predictability of post-TBI mortality and outcome, defined by the Glasgow Outcome Scale [4], using a core model of age, injury severity (motor subscale of the Glasgow Coma Scale [5]), and pupillary reactivity [6]. Further studies support this model by adding neuro-radiological findings, e.g., evidence of midline shift or presence of subarachnoid hemorrhage [7]. However, these models pertain to the outcome six months after the injury, predictors for later outcome are more diverse and result in conflicting results [8,9,10]. This may reflect the diversity of outcome measures and prognostic factors, which are often interdependent [10].

Although TBI produces direct, immediate damage (e.g., shearing and focal contusions), it is not a discrete event, it is an unfolding sequence of damage. During the acute event damaged neurons die, but also over the following days and weeks, additional cells die through cellular suicide (apoptosis) and oxidative stress [11]. The resultant secondary injury is associated with impairments of cerebral blood flow and metabolism, hypoxia, oedema, a cascade of neurochemical changes due to the release of excitotoxic neurotransmitters [12,13] and other dynamic factors (e.g., proinflammatory cytokines, brain-derived neurotrophic factor) influencing neuronal survival and synaptic plasticity [14,15]. Mechanisms of secondary injury influence the extent and type of impairments resulting from TBI, in the subacute and chronic period after injury (during inpatient rehabilitation), as well as during the acute phase. Mechanisms of secondary injury include multiple neurotransmitter alterations and cellular dysfunction [16]. For example, neuroendocrine disturbances along the hypothalamic-pituitary-adrenal axis can induce apoptosis [17]. Accordingly, patients suffering TBI with pituitary dysfunction were discharged from rehabilitation with a higher degree of disability, as measured by Functional Independence Measure and Disability Rating Score, than similar patients without pituitary dysfunction [18]. Moreover, the development of secondary complications such as hydrocephalus also plays a role in determining the course and outcome during and after rehabilitation [19]. Therefore, during rehabilitation, limiting and/or treating of late secondary injuries and medical complications is essential for improving long-term outcomes and limiting disabilities [19].

**Figure 1:**
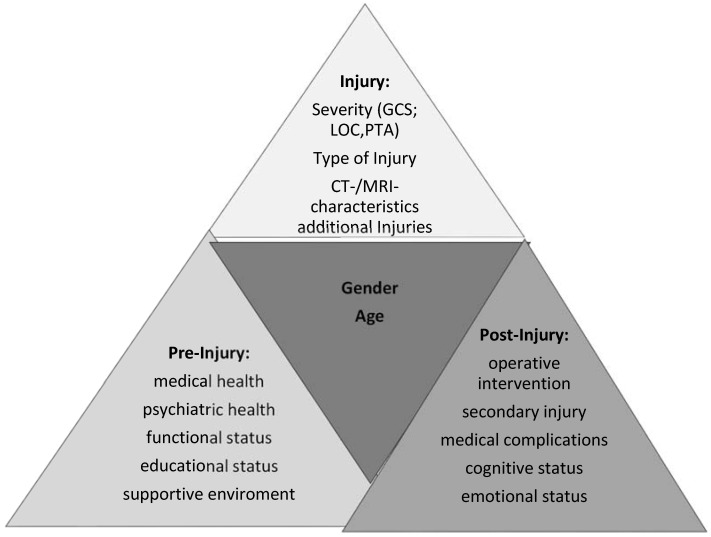
Interactivity between age and gender with pre-, post- and injury factors.

The functional outcome after TBI is related to a diversity of factors that can be divided into three broad categories: pre-injury factors, injury related factors (e.g., injury severity), and post-injury factors (*i.e.*, secondary injuries and medical complications) (Figure 1) [20,21,22]. Age is an important pre-injury related factor, and is a consistent determinant of TBI outcome. Older adults comprise a large segment of the population sustaining a TBI, with relatively worse outcomes than their younger counterparts when controlled for similar injury severity [15,23]. This may be caused not only by the increased premorbidity burden but also by the greater susceptibility in the older patients to secondary injury by excitotoxic neurotransmitters [24] and decreased neuronal survival [25]. This specifically illustrates the close interrelation between pre-injury status and injury and post-injury related factors. 

Gender is also a pre-injury factor that is related to injury, post-injury, and other pre-injury factors. Yet the literature on gender is discordant. Some studies have found greater mortality and morbidity related to gender, with women more vulnerable to greater damage [21,26]. Similarly there is some evidence of a greater tendency for increased intracranial pressure in pre-menopausal women, which would result in greater brain damage [27,28]. Davis and colleagues [29] reported better outcomes for post-menopausal women when compared with age-matched men, whereas pre-menopausal women showed no differences from men. Nevertheless there are studies suggesting younger women have less secondary injury and better outcomes, which have been attributed to neuroprotective effects of hormones for pre-menopausal women [24,30,31,32]. Other studies report no sex differences in acute complications or outcome [22,33,34]. Furthermore, reproductive function is down-regulated in episodes of severe illness, such as TBI [35]. Over the past 15 years researchers and clinicians have become increasingly aware of TBI-related hormonal disturbances [36,37,38,39]. Hypopituitarism often occurs in the post-acute phase of TBI and may normalize later; however, it may also develop after the post-acute phase [40].

Because endocrine impairments often include diffuse symptoms such as depression, cognitive decline, or fatigue, the treating clinician may attribute these symptoms to the typical sequelae of TBI or some complication encountered during rehabilitation instead of considering an endocrine dysfunction. 

Hence the present review seeks to examine the interrelation between neuroendocrine disturbances with medical complications frequently encountered after moderate to severe TBI during rehabilitation.

## 2. Neurorehabilitation

There is no clear, direct, and consistent relationship in TBI between pathology and impairment, both within a patient over time and between patients. For example, it is not uncommon to see patients with large areas of cerebral contusional bleeding but few symptoms, signs, or disabilities. At the same time patients may exhibit obvious clinical signs of functional impairment, while the traditional diagnostic studies may not show any tissue alteration [41]. Disabilities that may arise as a result of TBI often have physical, cognitive, behavioral, and psychosocial aspects (Table 1) [13,41]. TBI rehabilitation therefore requires comprehensive and individualized rehabilitation programs. The ultimate goal of TBI rehabilitation is to return the patient to his or her highest functional level by maximizing health and independence.

**Table 1:** Functional impairments frequently encountered after moderate to severe traumatic brain injury during rehabilitation.

**Motor Function**	Strength
	Coordination
	Dexterity
	Velocity of movement
	Ambulation
**Cognitive Function**	Alertness
	Attention
	Concentration
	Memory
	Learning
	Executive function
**Behavioural and Psychosocial Function**	Apathy
	Depression
	Anxiety
	Impulsivity
	Irritability
	Aggression/Agitation
	Social communication

A key property of the nervous system is its capacity to alter its structure and function in reaction to environmental change as well as to perturbations such as injury. Although the term plasticity is now widely used, it is not easily defined and is used to refer to changes at many levels in the nervous system ranging from molecular events, such as changes in gene expression, to behaviour [42]. Changes that occur after injury can be modified by many types of behavioural experiences (e.g., rehabilitation). The injury stimulates spontaneous reparative changes that interact with rehabilitative therapies [43]. Rehabilitation, including motor or cognitive exercise, can facilitate or augment neuroplasticity and functional recovery. On the other hand, later occurring medical complications, such as seizures, hydrocephalus, neuroendocrine disturbances (Table 2) can attenuate or even disrupt neuroplasticity, repair and recovery. Hence detection of signs and symptoms of neuroendocrine and medical complications and their timely treatment may prevent further secondary injury during rehabilitation.

**Table 2:** Overlap of clinical features frequently encountered after moderate to severe head injury with those following neuroendocrine disturbances (*i.e.*, anterior hypopituitarism).

Symptoms of Head Injury	Symptoms of Neuroendocrine Disturbances
Headache	ACTH: Headache, Nausea/vomiting/diarrhoea TSH: weight gain/constipation
Other chronic/acute pain	TSH: Arthralgia/myalgia ACTH: Stomach pain
	TSH: Cold intolerance
Sleep disturbance	Melatonin: Sleep disturbance
Visual impairment	
Vestibular impairment	
Dizziness	ACTH:Dizziness
Attention and/or concentration deficits	GH, testosterone, estrogen: Attention and/or concentration deficits Osteoporosis Decreased cardiovascular endurance Decreased lean body mass
Reduced libido	Testosterone, estrogen: Reduced libido
	Testosterone, estrogen, prolactin↑: Infertility Menstrual dysfunction Erectile dysfunction Decreased cardiovascular endurance (Osteoporosis)
Executive control deficits	GH: executive function deficits
Irritability	Estrogen: Irritability
Poor frustration tolerance	GH: Poor frustration tolerance
Anger	GH: Anger
Impulsivity	GH: Impulsivity
Lack of initiative, apathy	ACTH, GH, TSH, testosterone/estrogen: Lack of initiative, apathy
Loss of energy	ACTH, GH, TSH, testosterone/estrogen: Loss of energy
Getting tired easily	ACTH, GH, TSH, testosterone/estrogen: Getting tired easily
New learning and memory deficits	ACTH, GH, TSH, testosterone/estrogen: New learning and memory deficits
Feeling anxious	ACTH, GH, TSH, testosterone/estrogen: Feeling anxious
Feeling depressed	ACTH, GH, TSH, testosterone/estrogen: Feeling depressed

(ACTH: Adrenocorticotropin, GH: Growth Hormone, TSH: Thyroid Stimulating Hormone).

## 3. Neuroendocrine Disturbances

Any systemic illness or traumatic event may be associated with alterations in the hypothalamic-pituitary-peripheral hormone axes representing an adaptive response to a stressfull event [44]. The pituitary gland responds initially by increasing adrenocorticotropin (ACTH), growth hormone (GH) and prolactin secretion while decreasing or not altering the secretion of luteinizing hormone (LH), follicle-stimulating hormone (FSH) and thyrotropin [45]. These adaptations are protective at first, but if excessive or inadequate they may be associated with an increased morbidity. Thus when considering neuroendocrine disturbances following TBI, we have to distinguish between functionally adaptive alterations during the acute phase after TBI from increasing or decreasing pituitary hormone levels and the alterations in pituitary hormone secretion caused by damage to any level of the hypothalamic-pituitary circuit and that may occur at any time after TBI [44].

Since the 1940s, the literature has reported TBI as a source of neuroendocrine disturbances [46,47,48]. Roughly two-thirds of patients who died from severe head injury and had been autopsied were found to have structural irregularities in the hypothalamus, pituitary stalk, and/or pituitary gland [49,50,51]. Thus hormones produced or controlled along the pituitary axis can be dysregulated by TBI. The pituitary gland is particularly susceptible to mechanical and ischemic injuries (secondary to brain swelling or hypoxia) due to the vulnerability of its vascular supply through the *infundibulum* and firm encasement within the *sella turcica* [13,52]. Hypopituitarism is defined as a partial or complete loss of one or more of the hormones secreted by the pituitary gland [17]. The pituitary has two lobes, primary physical divisions each regulating distinct hormonal axes. The posterior lobe releases oxytocin and vasopressin. Vasopressin or antidiuretic hormone (ADH) is related to water retention and concentration of urine. An inappropriately high secretion leads to a concentrated urine and hypervolemic hyponatremia, while an insufficient secretion causes *diabetes insipidus* with a diluted urine, dehydration and/or hypernatremia [13]. The anterior lobe releases ACTH, GH, prolactin, TSH, follicle stimulating hormone (FSH) and lutenizing hormone (LH). These hormones interact with peripheral endocrine glands (e.g., adrenal cortex, liver, thyroid gland, gonads) and endocrine glands within the brain (e.g., hypothalamus and pineal gland). Typical generalized symptoms accompanying a deficiency of most of these hormones (Table 2) as fatigue, depression, anxiety, lack of energy, and cognitive dysfunction cannot be distinguished from the symptomatology frequently encountered after moderate to severe head injury [13,17]. 

Nevertheless there are specific symptoms in addition to the generalized symptoms characterizing pituitary insufficiency that should warrant further screening and/or tests during rehabilitation (Table 2). Adrenocortical insufficiency is the most potentially life threatening and severe syndrome; it can manifest early with dizziness, fatigue, nausea, vomiting, stomach pain, and diarrhea (autonomic dysfunction) and results in impairment of the body’s response to stress with hypoglycemia, weight loss *etc.* [13]. Hypothyroidism leads to cold intolerance, joint pain, constipation, weight gain, menstrual dysfunction and brittle hair [17]. Growth hormone deficiency is characterized by decreased lean body mass, increased abdominal fat due to an impaired lipid metabolism, osteoporosis, and decreased cardiac and cardiovascular function. Deficiencies along the gonadal axis in women exhibit menstrual dysfunction, reduced libido, infertility, irritability, depression, and in men exhibit reduced libido, infertility, depression, and reduced lean body mass. Hyperprolactinemia, a neuroendocrine disturbance with excess secretion of prolactin caused by stress and/or imbalance with catecholamines (dopamine) and serotonin (antipsychotic medication) can manifest with erectile dysfunction, infertility, or unwanted breast development in men, and with menstrual dysfunction and galactorrhea in women [17].

The incidence and prevalence of TBI-related hormonal disturbances are still unclear as different diagnostic and measurement criteria have been employed in various studies [36,53]. In addition the diagnosis of hypopituitarism after TBI can be confounded by the changes in circulating pituitary hormone levels caused by the adaptive response to critical illness and/or pharmacological therapy (e.g., dopaminergic agents, glucocorticoids) [45]. In the last few years, a number of systematic studies have shown a decline in sex hormone and insulin-like growth factor 1 (IGF-I) levels in the acute phase after trauma [40,53,54]. Hypopituitarism often occurs in the post-acute phase of TBI and may normalize later; however, it may also develop after the post-acute phase. Schneider and colleagues described the prevalence of anterior pituitary insufficiency at 3 months (56% of all patients) and 12 months (36% of all patients) after TBI [40]. At three months, the extent of hypogonadism was directly proportional to the severity of the traumatic brain injury [40]. Agha and colleagues evaluated the prevalence of anterior and posterior pituitary dysfunction in the early phase after TBI [54]. 80% of patients had gonadotropin deficiency.

Patients can also develop neuroendocrine disturbances in the post-acute and chronic phases. In a prospective study by Tanriverdi and colleagues covering five years, GH deficiency was found to be the most common pituitary hormone deficit at one, three, and five years following TBI [55]. They also reported that most of the pituitary hormone deficiencies improved over five years, but a substantial number of patients still had a deficiency of one hormone at the fifth year. Some patients, although rarely, may develop new onset hypopituitarism, or their pituitary dysfunction may worsen over the years [56]. Screening has become more frequent with increased awareness due to reports in the literature. Lauzier and his colleagues recently reviewed 66 studies with 5386 patients: over the long term, 31.6% of patients had at least one pituitary hormone deficiency [39]. They and others also reported that age, severe head injury, and skull fractures were associated with an increased risk of anterior pituitary disorders [39,56].

Post-traumatic pituitary insufficiency may contribute substantially to morbidity and mortality in TBI patients. Although the review by Lauzier *et al.* found no difference in clinical outcome measured by the Glasgow outcome scale for patients with anterior pituitary dysfunction, they also noted the significant heterogeneity among the studies reviewed and that only a few studies evaluated actual neurologic outcomes [39]. Other authors using more sensitive outcome variables such as the Functional Independence Measure or the Cognitive Functioning Scale reported worse outcome measures [18] and worse cognitive function in patients with pituitary insufficiency after sustaining TBI than their counterparts without pituitary insufficiency [57]. In addition, there is ample evidence that the treatment of hypopituitarism leads to improvements in medical, behavioural, and cognitive function [58]. The Consensus guidelines for screening of neuroendocrine function after TBI recommend hormonal screening as clinically indicated during the acute hospital stay and again at 3 and 12 months post injury [37]. Current recommendations involve screening all moderate to severe head injury patients beginning with testing for hypocortisolism with a morning cortisol level within seven days post injury [13]. Nevertheless, during the acute phase the adaptive response to critical illness may still prevail, therefore screening should be repeated to exclude the potentially life threatening secondary adrenal insufficiency [45]. Pertaining to the time in rehabilitation, there should be screenings at three and six months post injury including tests of free T4, TSH, IGF-1, testosterone, estradiol, LH, and FSH. GH testing requires consultation with an endocrinologist, as it involves special dynamic testing [13]. Formal testing is critical as clinically diagnosing neuroendocrine dysfunctions is difficult considering the nonspecific symptomatology following TBI. (Table 2) [17,58]. Patients who develop diabetes insipidus, adrenal insufficiency, or other symptoms of hypopituitarism should undergo testing of the entire pituitary axis without waiting for three months. For patients more than 12 months post-TBI who have not had previous screening, a baseline hormonal workup is recommended [58]. Neuroendocrine disturbances can also develop as late as five years after the injury caused by the development of anti-hypothalamic or anti-pituitary antibodies rather than due to the initial injury to the pituitary [56]. Therefore patients with worsening symptoms, e.g., fatigue, depression, cognitive dysfunction, over some years may profit from testing anew for pituitary insufficiency. Moreover the presence of neuroendocrine abnormalities may be masked by the occurrence of medical complications frequently encountered during rehabilitation or they in turn can aggravate the course of medical complications. The need for monitoring for development of pituitary insufficiency after TBI in order to avoid long-term adverse consequences was emphatically stated in the 2009 Institute of Medicine report on the Gulf War [59]. The sections below highlight the possible interactions of neuroendocrine disturbance and typical complications following moderate to severe head injury.

## 4. Medical Complications

### 4.1. Hydrocephalus

Hydrocephalus is a common complication after TBI. The incidence of hydrocephalus varies from 5%–45%, depending on the severity and the setting (acute *versus* post-acute) of the study [60,61]. Tian and colleagues reviewed the incidence of posttraumatic hydrocephalus in patients with traumatic subarachnoid hemorrhage and reported that 12% of patients with traumatic subarachnoid hemorrhage developed hydrocephalus within three months [62]. Kammersgaard and colleagues investigated the incidence of hydrocephalus for patients (*n* = 444) requiring rehabilitation in Denmark [63]. They observed an incidence of 14% with three-quarters occurring during rehabilitation. Hydrocephalus is defined as “an active distention of the ventricular system of the brain related to inadequate passage of cerebrospinal fluid from its production within the ventricular system to its point of absorption in the systemic circulation” [64].

Risk factors for post-traumatic hydrocephalus include advanced age, injury severity, intraventricular haemorrhage, subarachnoid haemorrhage, meningitis, the use of decompressive craniotomy, and the duration of coma [61,65]. However, Kammersgaard did not find an increased incidence of hydrocephalus after decompressive craniotomy [63]. Patients with post-traumatic hydrocephalus were older, had a higher injury severity (measured by Glasgow coma scale at admission to rehabilitation) and had a low level of consciousness. 

Post-traumatic hydrocephalus is the most common treatable neurosurgical complication during rehabilitation [66]. There are two types of hydrocephalus: communicating and non-communicating hydrocephalus. Communicating hydrocephalus is more frequent after TBI, and is caused by disruption of the absorptive capability of the arachnoid villae. Different portions of the ventricular system can “communicate” with each other, and cerebrospinal fluid may flow from the ventricles to the subarachnoid space. Blood products and/or inflammatory products impede the flow of the cerebrospinal fluid through the arachnoid granulations into the blood stream causing a gradual enlargement of the ventricles. It also known as normal pressure hydrocephalus as the intracranial pressure is only intermittently elevated, but the enlarged ventricles put increased pressure on the adjacent cortical tissue [64].

Post-traumatic hydrocephalus can cause myriad symptoms in the patient: the typical clinical triad for normal pressure hydrocephalus is gait ataxia or short-stepping gait, cognitive decline, and urinary incontinence or urge. Usually the onset is slow, and a high index of suspicion is required of the treating physician. The earliest indication is often a decline or plateau in functioning and/or subtle mental status changes [67]. The symptoms of a slow decline or plateau of functioning are also seen due to *neuroendocrine disturbances*: e.g., hyponatremia due to inadequate ADH secretion, anterior pituitary insufficiency. Also the later symptoms of hydrocephalus, indicative of increased intracranial pressure, such as headache, nausea, vomiting and lethargy, coincide with symptoms signalling ACTH deficiency (Table 3). The diagnosis is not always simple; most physicians rely on a combination of imaging criteria, other measurements of cerebral spinal fluid dynamics, and clinical signs.

**Table 3:** Medical complications frequently encountered after moderate to severe head injury during rehabilitation, their symptomatology, and risk factors.

Medical Complications	Symptomatology	Risk Factors	Neuroendocrine Disturbance Causing Similar Symptoms
Neuroendocrine disturbances		advanced age, injury severity, skull fractures	
Hydrocephalus	(1) Reduced functioning, lethargy, nausea, vomiting (2) Gait disturbance (3) Urinary incontinence	advanced age, injury severity, intraventricular haemorrhage, subarachnoid haemorrhage, meningitis	(1) Anterior pituitary insufficiency: GH, TSH, ACTH
Post-traumatic epilepsy	(1) Seizures, (2) reduced cognitive performance, including alertness and speed of processing	skull fractures, penetrating injury, advanced age, neurological deficit	(1) hypo- or hypernatremia due to SIADH or Diabetes insipidus can trigger seizures (2) Anterior pituitary insufficiency: GH, TSH
Fatigue	anxiety, depression daytime sleepiness, diminished cognitive function	gender (female)? Pituitary dysfunction? anxiety, depression, sleep disturbances, cognitive and motor disturbance, pain	Anterior pituitary insufficiency: GH, TSH, ACTH Decreased evening melatonin synthesis (sleep disturbance)
Disorders of consciousness		Injury severity	?
Paroxysmal sympathetic hyperactivity	(1) ↑ Heart rate (2) ↑ Blood pressure (3) ↑ Respiratory rate (4) Sweating (5) ↑ Temperature (6) Posturing (7) ↑ reactivity to a non-noxious stimulus	injury severity diffuse axonal injury gender (male), younger age	
Psychiatric-behavioural Symptoms (Apathy, Depression, Anxiety, Agitation/Aggression)	(1) Apathy (2) Depression (3) Anxiety (4) Agitation/Aggression	(1) focal brain injury (frontal) (2) younger age, pre-injury mental health treatment, pre-injury substance abuse, gender (female) (3) older age (4) Injury severity, focal brain injury (frontotemporal) Gender (male)	(1) Anterior pituitary insufficiency: GH, TSH, ACTH (2) Anterior pituitary insufficiency: GH, TSH, ACTH (3) Anterior pituitary insufficiency: GH, TSH, ACTH (4) ACTH Insufficiency

Early diagnosis and treatment (*i.e.*, shunt implantation) are important to prevent worsening of the above mentioned signs and symptoms or, even worse, alterations of consciousness and global and/or irreversible cognitive dysfunction. Should symptoms persist even after shunt implantation, the clinician should consider neuroendocrine disturbance in his differential diagnosis.

### 4.2. Epilepsy

The presence of post-traumatic seizures has a significant influence on outcome, including medication use, quality of life, employment, and psychosocial adjustment. Post-traumatic epilepsy is defined as two or more unprovoked seizures that occurred at least seven days after TBI. Post-traumatic seizures (PTS) can be classified into three categories: immediate PTS (within 24 hours), early PTS (24 hours-7 days) and late PTS (post seven days) [68,69]. The incidence of early seizures is reported to range between 3% and 16% of patients, depending on the study design [68,70]. The development of early and not immediate seizures may predispose to the development of late seizures; as a consequence the Brain Trauma Foundation and the American Academy of Neurology recommend prophylaxis with anti-epileptic drugs for seven days after TBI to prevent late seizures [69,71]. This recommendation is controversial in the literature. Some studies of prophylactic treatment with phenytoin report a higher incidence of fever [72] or worse functional outcome [73]; others recommend abiding by the guidelines of the Brain Trauma Foundation [74] and report an equivalent effect of phenytoin and levitiracetam in the early prophylaxis [75]. The pathophysiology leading to the occurrence of early seizures is probably multifactorial, including the interruption of the blood-brain-barrier, the presence of blood and the injury-related release of excitotoxic neurotransmitters [76,77]. Risk factors for early seizures include severe head injury, immediate seizures, depressed skull fractures, penetrating head injury, cerebral contusions, intracranial haemorrhage, chronic alcoholism and age older than [71,76,77].

The reported incidence of late seizures depends on the study design. Although onset of post-traumatic seizures is commonly observed in the first year post injury, an increased risk of late-onset seizure activity continues for several years [78]. In a population-based cohort study by Annergers, the incidence of late seizures after severe head injury was 7% after one year and 12% after five years [68]. In a recent study about the prevalence of post-traumatic epilepsy in veterans of the Afghanistan and Iraq wars, the incidence observed was 11%, and 80% of the PTS occurred within two years after the injury [79]. The presence of skull fractures, penetrating injury, increased age at the time of injury, and neurological deficit predispose to the development of PTS [79]. The necessity to treat PTS is based on the knowledge that patients with PTS have an additional disadvantage regarding ongoing physical, cognitive, psychosocial, and reintegration issues following brain injury compared to patients without PTS [80,81]. Additionally, there is evidence that interictal epileptiform discharges and/or seizures affect cognitive performance, including alertness and speed of processing, at the moment of their occurrence, but also contribute to negative long-term cognitive outcomes such as decreased educational achievement [82,83]. Further it is of the utmost importance to control and/or regulate sodium and glucose levels to avoid hypo- and/or hyper-natremia as well as hypo- and/or hyper-glycemia, as these are well known to provoke seizures. These imbalances may be produced by neuroendocrine disturbances (Table 3).

### 4.3. Fatigue

Fatigue is a common, heterogeneous, and disabling complaint of patients who have sustained a moderate to severe brain injury, with a multifactorial aetiology. Sleep disturbances commonly follow TBI and may contribute to fatigue. Shekleton and colleagues found reduced evening melatonin levels in TBI patients indicating a disruption to circadian regulation of pineal melatonin [84]. Both brain pathology and secondary factors are implicated. Depression, anxiety, and pain exacerbate fatigue [85]. Its prevalence has been reported to be 16%–80% of individuals after TBI, depending on the time since injury and the measure used to quantify it [86,87]. The measurement of fatigue used in studies is heterogeneous and an accurate measurement seems challenging given the interrelatedness of fatigue and daytime sleepiness and mood disturbances [88]. Five scales have been used to evaluate fatigue in TBI patients: the Fatigue Severity Scale, the visual analog scale for fatigue, the Fatigue Impact Scale, the Barrow Neurological Institute Fatigue Scale and the Cause of Fatigue Questionnaire. The Barrow Neurological Institute Fatigue Scale and the Cause of Fatigue Questionnaire have been designed specifically for brain-injured patients [89]. Fatigue is associated with anxiety, depression, sleep disturbances, cognitive and motor disturbance and pain [90]. Ponsford and her group even hypothesizes that fatigue after TBI is a cause and not a consequence of anxiety, depression, and daytime sleepiness, while depression may exacerbate fatigue by diminishing cognitive function [91]. Sleep disturbances contribute to fatigue. Objective sleep studies show reduced sleep efficiency, increased sleep onset latency, and increased time awake after sleep onset and increased slow-wave sleep. Individuals with TBI show lower levels of evening melatonin production, associated with less rapid-eye movement sleep [88]. However, fatigue is also related to slowed information processing, reduced psychomotor performance, and the need for increased effort in performing tasks therefore hindering rehabilitation [88,92]. Potential medical causes such as endocrine dysfunction and anemia should be considered before beginning any treatment, although Englander and his colleagues reported no correlation between pituitary dysfunction and fatigue in a sample of 119 patients [85]. They further concluded that fatigue most robustly correlated with depression, pain, and self-assessment of memory and motor dysfunction. However Bushnik examined 64 individuals for more than one year post-TBI and found a trend between lower basal cortisol and greater fatigue [93]. Nevertheless, considering many reports about the presence of fatigue caused by cortisol, thyroid, or GH deficiency [13,94], it seems reasonable to exclude neuroendocrine disturbances before starting any treatment. Taking into consideration the Ponsford model of fatigue, with fatigue being the cause of anxiety and depression, this may be even more important, as depression and anxiety can also be the consequence of neuroendocrine disturbance (Table 2).

### 4.4. Disorders of Consciousness

Patients with severe head injuries often experience prolonged disorders of consciousness and pass through different levels of consciousness. Patients sometimes remain in states such as the unresponsive wakefulness syndrome [95] or the minimally conscious state [96]. Patients with unresponsive wakefulness syndrome show no signs of awareness of themselves or their environment. Still, they open their eyes spontaneously and have sleep–wakefulness cycles [97,98]. Minimally conscious patients show inconsistent signs of awareness, but are usually able to fixate or to follow simple commands.

In the past, disorders of consciousness were considered to be associated with a poor prognosis and therefore thought of as contraindications for acute inpatient rehabilitation [99,100]. Recent literature regarding the natural history of disorders of consciousness shows a more favourable prognosis in terms of both recovery of consciousness and recovery of functional independence [101,102]. Especially long-term observations show a substantial minority of patients improve in function [103,104]. Considering that patients with disorders of consciousness have a higher incidence of medical complications [105], re-hospitalisations [106], and that their outcome is negatively correlated with the number of complications experienced [107], it seems especially important to screen these patients periodically for neuroendocrine disturbances. These patients cannot communicate their symptoms. To this point, patients with prolonged disorders of consciousness have not been included in studies of neuroendocrine disturbances as neurocognitive dysfunctions cannot be observed or tested. Conceivably, hormonal imbalance may contribute to disorders of consciousness. There is only one study that examined neuroendocrine disturbances prospectively over two years in patients with severe head injuries and found an association between hormonal disturbances during the acute phase and prolonged mechanical ventilation [108]. Inherently, many patients with disorders of consciousness are mechanically ventilated over a long time, therefore testing for neuroendocrine disturbances may be valuable. Prospective studies will be required to examine neuroendocrine disturbances in patients with prolonged disorders of consciousness.

A very recent study by Steppacher and colleagues casts doubts on the 12-month boundary, after which the unresponsive wakefulness syndrome is considered to be permanent [109]. They observed 102 patients with disorder of consciousness over 2–14 years after the event and found 30 of them regained consciousness and developed some communicative ability. Six patients regained consciousness after more than three years, and the length of the disorder of consciousness did not predict the clinical outcome.

### 4.5. Paroxysmal Sympathetic Hyperactivity

Patients with moderate to severe brain injury commonly demonstrate short-term elevations of autonomic parameters in the ICU. A subgroup of these patients goes on to develop a syndrome of persistent paroxysmal sympathetic and motor hyperactivity (PHS). This syndrome is also known by various other names including dysautonomia, paroxysmal sympathetic storms, or paroxysmal autonomic instability with dystonia [13]. PHS can arise from many types of acquired brain injury, but the majority of published cases resulted from traumatic brain injury with 79% followed by hypoxia with 10% and stroke with 5% [110]. All other etiologies are rare. The possible association between lesion location and the development of PSH studies is controversial. Some report a tendency toward focal lesions [111], others report a significant presence of diffuse lesions to be present in PSH [112]. Zang and colleagues suggest a certain degree of diffuse injury to be present in all TBI patients regardless of the presence of focal structural lesions [113]. The underlying pathophysiology is still unknown and the most recent excitatory:inhibitory model proposed by Baguley finds wide acceptance: dysfunction of the diencephalic-brainstem inhibitory centre that normally controls afferent stimulus processing in the spinal cord is thought to result from functional disconnections related to the traumatic damage of deep brain structures. As a result there is unopposed adrenergic outflow with increased levels of circulating catecholamines [112].

Recognition of the syndrome is important because it is common, affecting 8%–10% of patients with severe head injury [111,114] and associated with increased morbidity [110]. It is associated with longer hospitalizations and because of the increased sympathetic tone with hypermetabolism and weight loss, cardiac dysfunction, immune suppression, increased incidence of heterotropic ossification, increased likelihood of tracheostomy, worse outcome by Functional Independence Measure, increased duration of posttraumatic amnesia [110]. PSH represents a potentially treatable contributor of secondary brain injury. Therefore a timely diagnosis and treatment are important to improve functional outcome [115].

The International Brain Injury Association has recently convened a consensus workgroup to clarify the nomenclature and diagnostic criteria for this syndrome. The proposed term from this consensus group is paroxysmal sympathetic hyperactivity [116]. PSH is a syndrome that does not differ based on the underlying etiology and occurs across a spectrum of severity. It is identified by simultaneous, paroxysmal transient increases in sympathetic (heart rate, blood pressure, respiratory rate, temperature, sweating) and motor (posturing) activity. Further clinical items to determine the diagnosis are for example persistence of these features ≥3 consecutive days, the occurrence of these features also following normally non-painful stimuli, the occurrence of these features ≥2 weeks post injury with a frequency of ≥2 episodes daily. Few patients with PSH exhibit all features [116]. The diagnosis is one of exclusion and alternative diagnoses have to be excluded, such as opiate or sedation withdrawal, systemic inflammatory response syndrome, or painful spasticity/dystonia or overlap syndromes (e.g., neuroleptic malignant syndrome).

Thus far there is no literature investigating the hypothalamic and/or pituitary axes and their hormones in patients with prolonged disorders of consciousness and PSH. One may assume that a hyperactive sympathetic state precludes pituitary insufficiency, but it has not been examined. Possibly, an increased secretion of corticotropin releasing factor in the presence of a decreased secretion of ACTH may cause this hyperadrenergic stress response. Prospective studies will be needed to unravel the interrelation of PSH, another neuroendocrine disturbance and hypothalamic-pituitary function.

### 4.6. Psychiatric-Behavioural Symptoms (Apathy, Depression, Anxiety, Agitation/Aggression)

Mood problems, depression, anxiety agitation, and aggression have been reported to follow moderate to severe TBI. Mood and behavioural disorders interfere with rehabilitation and can result in repeated hospitalisations, alienation from family and friends, and unemployment [117]. Discerning whether psychiatric symptoms are due to the direct injury to the brain or to the patient’s reaction to the injury, the TBI-related deficits or inadequate coping strategies, is hardly possible [17].

The most frequent neuropsychiatric disorder observed by Ciurli and his group in patients with severe TBI using the Neuropsychiatric Inventory was apathy in 42% of cases, followed by irritability (37%), depressed mood (29%), and agitation/aggression (24%) [117]. Apathy was not correlated with injury severity but with focal brain injury, especially in the orbitofrontal regions. In the acute recovery period, agitated behaviour (ranging from restlessness to overt aggressiveness) is frequently reported [118]. It usually resolves prior to the resolution of posttraumatic amnesia but can also continue into the chronic phase and impede rehabilitative efforts and community integration [119]. Agitation and restlessness in the acute phase are also associated with more severe head injuries, focal lesions mainly localized in the frontotemporal region [120] and with residual emotional and cognitive impairments one year after injury. Owing to different methodologies and assessments of depression, there are variable prevalence estimates of depression in patients with TBI reported in the literature [121]. A recent meta-analysis found that 27% of people were diagnosed with depression following TBI and 38% reported clinically significant levels of depression when assessed with self-report scales. Estimates of depression varied according to diagnostic criteria (ICD-10: 14%; DSM-IV: 25%; DSM-III: 47%) and injury severity (mild: 16%; severe: 30%) [121]. Both minor and major depression diagnosed during rehabilitation are associated younger age, pre-injury mental health treatment, and substance abuse [122]. Although minor depression is less common (22%) than major depression (26%) it is also correlated with negative outcomes related to participation and quality of life [122]. In a longitudinal study of minor and major depression one to two years post TBI, Hart and colleagues observed almost one third of those with minor depression during the first year after TBI traversed to major depression in the second year and worsening was associated with lower cognitive function, poor social support, and pre-injury mental health issues including substance abuse [123]. These results highlight the importance of long-term monitoring for depression and treating minor as well as major depression.

All these psychiatric symptoms may also signal endocrine dysfunctions, especially depression, anxiety, and apathy are a typical non-specific signs of anterior pituitary insufficiency [17]. Agitation and aggression have also been reported to occur in a case of adrenal insufficiency following severe TBI [124]. The initiation of this patient’s treatment with prednisone and fludrocortisone allowed his full participation in a comprehensive rehabilitation program, which led to an excellent functional recovery including return to work and resumption of appropriate social and leisure activities.

Therefore the presence or new occurrence of psychiatric symptoms justifies testing for neuroendocrine disturbances, as the treatment for depression or aggression caused by neuroendocrine disturbances differs from the symptomatic treatment of depression.

## 5. Gender

Gender is a subject associated pre-injury factor that is intertwined with injury related, post-injury and other pre-injury factors. Some investigations reported sex differences in clinical response to TBI [125], but the data is contradictory [22]. Although brain structure, size, and function are affected by the person’s sex [126], one has to keep in mind that after having suffered TBI the hormonal milieu is altered due to functionally adaptive hormonal alterations in the hypothalamic-pituitary-peripheral axes during the acute phase [44] and possibly due to the alterations in pituitary hormone secretion caused by damage to any level of the hypothalamic-pituitary circuit that may occur at any time thereafter [45]. A very recent meta-analysis considering a person’s gender and age as a kind of biological or neurological reserve, examined sex difference in outcome [127]. They used 18 studies with various outcome measures and found no significant differences when outcome measures where combined. Although sex differences were evident for some outcome measures, it is possible that these differences were present prior to injury and did not reflect differential responses to TBI [127]. Reviewing the literature for sex differences pertaining the occurrence of frequently encountered complications following TBI, there was no association of gender with an increased risk of developing neuroendocrine disturbances [22,39,53], posttraumatic hydrocephalus [63], or recovery from prolonged disorders of consciousness [109]. There was also no clear evidence for gender being associated with a higher risk to develop PTS, except for one study by Cancelliere and colleagues, who reported an increase risk for females (children, young adults) to develop PTS after mild TBI [128].

However, Engländer [85] reported the incidence of fatigue correlated with female gender, but he used two types of fatigue measurement scales and gender correlated only when using two of the subtests of Multidimensional Assessment of Fatigue Scale and not with the Fatigue Severity Scale. PSH was more frequently described in younger patients and in males [111].

Regarding psychiatric-behavioural symptoms there might be association with gender: apathy was not correlated with gender [117], but both minor and major depression were associated with female gender [122]. Male veterans after TBI reported increased aggression compared to female veterans, these differences may be related to the interaction of TBI with sex-specific patterns of brain connectivity [129]. 

Overall men and women experience broadly comparable complications and outcomes with some exceptions, e.g., the psychiatric- behavioural symptoms. These exceptions should be considered during rehabilitation.

## 6. Conclusions

Traumatic brain injury is not just a discrete event, but an unfolding sequence of damage. Additional secondary injury can ensue due to neuroendocrine disturbances and/or medical complications not only during the acute phase but also in the subacute and chronic period after injury, *i.e.*, during inpatient rehabilitation. Thus, functional outcome after moderate and severe TBI is highly variable and related to a diversity of subject associated factors. Neurorehabilitation, for example motor and cognitive exercise, can positively influence functional recovery by enhancing neuroplasticity, can facilitate or augment neuroplasticity, and minimize further injury through recognition and treatment of neuroendocrine disturbances and medical complications. Neuroendocrine disturbances are easily overlooked as they often present with diffuse symptoms such as fatigue, depression, poor concentration, or a decline in overall cognitive function, typical sequelae of traumatic brain injury. Furthermore, medical complications such as hydrocephalus, epilepsy, fatigue, disorders of consciousness, paroxysmal sympathetic hyperactivity, or psychiatric-behavioural symptoms may mask and/or complicate the diagnosis of neuroendocrine disorders following TBI and may delay appropriate diagnosis, treatment, and impede neurorehabilitation. On the other hand, neuroendocrine disturbances along the hypothalamic-pituitary-adrenal axis (e.g., increased secretion of ACTH) also occur as sequelae of experienced stress due to helplessness, depression, social isolation, and can lead to cell death in hippocampus, amygdala, cerebellum and medial prefrontal cortex [17]. This reflects the interaction and interdependence of hormones with cognitive and behavioural outcomes and emphasizes the purpose of neurorehabilitation, to maximize functional outcome and independence and to minimize further injury.

The complex interaction and influence of hormones is also reflected in the ambiguous literature about the protective effects of steroids. Although early administration of progesterone showed neuroprotective effects in animal studies [130,131], these effects were not replicated in the recent studies by Wright and colleagues or by Skolnick and colleagues [132,133]. Correspondingly, the protective effect of sex steroids seen in animal studies seems debatable. Female gender as a risk factor for depression and fatigue after TBI has been reported, male gender as a risk factor for PSH and aggression, while hydrocephalus, posttraumatic epilepsy, and disorders of consciousness were not associated with gender. Wagner and her group even reported worse outcomes for patients with extended serum elevations of estradiol and testosterone levels in the acute phase, but gender did not affect outcome [134]. Yet she later conveyed that higher estradiol/testosterone ratios in the cerebral spinal fluid were associated with better outcomes [135]. The complex central and peripheral interactions of hormones and their disturbances may offer one of many explanations for the variability in outcome and complications after moderate to severe TBI and further studies monitoring hormones in serum and the cerebral spinal fluid are needed.
